# Unveiling the Role of Tumor-Infiltrating T Cells and Immunotherapy in Hepatocellular Carcinoma: A Comprehensive Review

**DOI:** 10.3390/cancers15205046

**Published:** 2023-10-19

**Authors:** Xiaokun Chen, Xiao Liu, Shunda Du

**Affiliations:** 1 Department of Liver Surgery, Peking Union Medical College Hospital, Peking Union Medical College and Chinese Academy of Medical Sciences, Beijing 100730, China; pumchchenxiaokun@icloud.com (X.C.); radray@126.com (X.L.); 2 Graduate School, Peking Union Medical College and Chinese Academy of Medical Sciences, Beijing 100730, China

**Keywords:** tumor-infiltrating T cells, immunotherapy, hepatocellular carcinoma, tumor microenvironment

## Abstract

**Simple Summary:**

Hepatocellular carcinoma (HCC) is a pressing global health concern, ranking third in cancer-related deaths. Current treatments have limited success, with low survival rates and high recurrence. Immunotherapies show promise in improving outcomes, such as combining atezolizumab and bevacizumab. However, more effective treatments are needed. This review explores the immune landscape of HCC, shedding light on the complex interactions among T cells within the tumor environment and strategies to reinvigorate these cells. It also summarizes ongoing trials of immune checkpoint inhibitors, combination therapies, and CAR-T or TCR-T cell therapies for HCC, which may transform its management.

**Abstract:**

Hepatocellular carcinoma (HCC) is a rapidly rising global health concern, ranking as the third-leading cause of cancer-related mortality. Despite medical advancements, the five-year survival rate remains a dismal 18%, with a daunting 70% recurrence rate within a five-year period. Current systematic treatments, including first-line sorafenib, yield an overall response rate (ORR) below 10%. In contrast, immunotherapies have shown promise by improving ORR to approximately 30%. The IMbravel150 clinical trial demonstrates that combining atezolizumab and bevacizumab surpasses sorafenib in terms of median progression-free survival (PFS) and overall survival (OS). However, the therapeutic efficacy for HCC patients remains unsatisfactory, highlighting the urgent need for a comprehensive understanding of antitumor responses and immune evasion mechanisms in HCC. In this context, understanding the immune landscape of HCC is of paramount importance. Tumor-infiltrating T cells, including cytotoxic T cells, regulatory T cells, and natural killer T cells, are key components in the antitumor immune response. This review aims to shed light on their intricate interactions within the immunosuppressive tumor microenvironment and explores potential strategies for revitalizing dysfunctional T cells. Additionally, current immune checkpoint inhibitor (ICI)-based trials, ICI-based combination therapies, and CAR-T- or TCR-T-cell therapies for HCC are summarized, which might further improve OS and transform the management of HCC in the future.

## 1. Introduction

The global incidence of liver cancer was projected to reach approximately 906,000 cases in the year 2020, with hepatocellular carcinoma (HCC) emerging as the predominant histological subtype [1]. HCC, characterized by a five-year survival rate of only 18%, stands as the third-leading cause of cancer-related deaths worldwide. The notably grim prognosis linked to HCC presents a significant and complex healthcare challenge of global significance [2].

For the majority of HCC patients, the prevailing backdrop involves a chronic inflammatory environment within the liver. This inflammation arises from persistent viral infections such as hepatitis B viruses (HBV) and hepatitis C viruses (HCV), as well as conditions like alcoholic steatohepatitis (ASH) and non-alcoholic steatohepatitis (NASH). This intricate landscape is further complicated by factors such as metabolic syndrome, tobacco use, and even rare monogenic disorders, all of which have been linked to an elevated susceptibility to HCC. The introduction of HBV vaccination and the availability of potent antiviral therapeutics has ushered in a decline in the global incidence of HBV/HCV-associated HCC since the early 2000s [3,4,5]. In contrast, there has been a noticeable rise in HCC cases stemming from non-alcoholic fatty liver disease (NAFLD) and its severe variant, NASH [6].

Based on the Barcelona Clinic Liver Cancer (BCLC) staging system, which is a comprehensive tool used to categorize HCC based on tumor characteristics, liver function, and the patient’s overall health status [7] and in accordance with international guidelines, surgical intervention stands as the primary treatment approach for early-stage HCC, specifically those falling into stage 0/A [8]. However, it is important to note that despite these interventions, the recurrence rate within a five-year period remains alarmingly high, reaching up to 70% [9]. For the substantial majority of HCC patients classified under stage B/C, a range of treatment options are available, including transarterial chemoembolization (TACE), ablation therapy, systemic treatment, and immune therapy [10,11,12]. Despite the various therapeutic approaches, the median overall survival (mOS) outcomes for these stages have been underwhelming. Notably, immune checkpoint blockade (ICB) therapies, such as anti-PD-1/L1 and anti-CTLA4 antibodies, have emerged as a promising alternative therapy for HCC. Despite extensive efforts, the therapeutic efficacy for patients with HCC remains suboptimal, often hindered by treatment resistance. It is important to note that the overall response rate (ORR) to these interventions remains below 30%, highlighting the need for further advancements in uncovering the mechanisms of the tumor microenvironment (TME). In this review, our aim is to comprehensively explore the TME in HCC. We will delve into the specific roles of T cells in the mechanisms against tumors. Furthermore, we will discuss the most recent advancements in treatment strategies that focus on revitalizing impaired T cells within the HCC setting.

## 2. The Tumor Microenvironment of HCC

The liver is widely recognized for its ability to effectively regulate immune responses to dietary antigens, microbial agents, and various environmental stimuli. Within this immune-regulatory framework, T cells expressing PD-L1 play a critical role in maintaining hepatic immune tolerance. They do so by interacting with PD-1, which in turn curbs the activity of nearby T cells. The liver accommodates a significant number of immune checkpoints, along with tissue-resident macrophages and lymphocytes. This collective presence establishes a diverse range of mechanisms that promote immune tolerance within the liver’s microenvironment [13]. These mechanisms play a crucial role in constraining the progression of chronic liver diseases, including cirrhosis and hepatocellular carcinomas, by preventing unnecessary immune responses and maintaining tissue homeostasis. However, when liver injury occurs, often triggered by viral infections or chronic hepatitis, a cascade of events ensues, characterized by chronic inflammation and the subsequent accumulation of immune cells within the liver. This persistent inflammation, in turn, triggers the activation of hepatic stellate cells (HSCs), which play a pivotal role in liver fibrosis. Importantly, activated HSCs contribute to the excessive production of extracellular matrix (ECM) components, leading to the formation of scar tissue. In a study by Giraud et al. [13], single-cell RNA sequencing (scRNA-seq) was utilized to unveil the early expansion of TREM2+ CD9+ scar-associated macrophages and scar-associated mesenchymal cells during the progression of liver cirrhosis.

**Figure 1:**
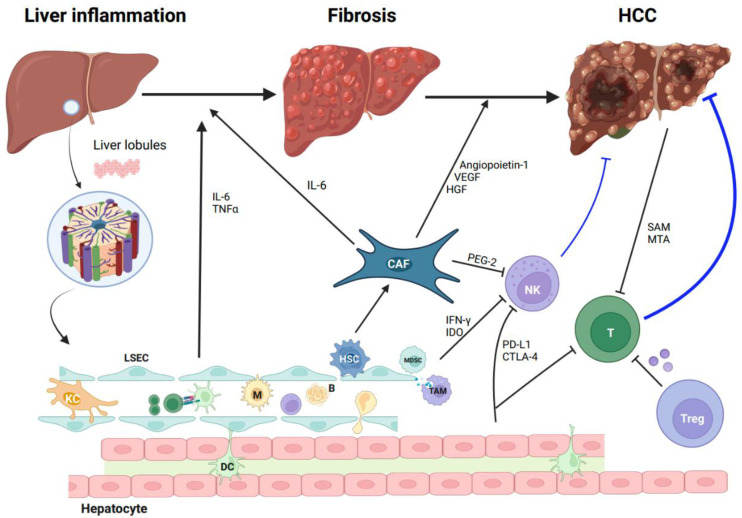
The microenvironment landscape of HCC development. The intricate interplay between immune cells and non-immune constituents plays a pivotal role in the progression of HCC. Liver sinusoidal endothelial cells (LSECs) have been implicated in tumor immune evasion and tumorigenesis through the release of IL-6 and TNFα. Cancer-associated fibroblasts (CAFs), originating from hepatic stellate cells (HSCs), significantly contribute to tumor-promoting inflammation by secreting elevated levels of IL-6, hepatocyte growth factor (HGF), vascular endothelial growth factor (VEGF), and angiopoietin-1. Various cellular components within the tumor microenvironment (TME), including CAFs, myeloid-derived suppressor cells (MDSCs), and tumor-associated macrophages (TAMs), produce immunosuppressive factors such as PGE2, IDO enzymes, and IFN-γ, actively hindering NK cell activation and cytotoxicity. Additionally, HCC-released metabolites, such as S-adenosyl-L-methionine (SAM) and methylthioadenosine (MTA), induce chromatin accessibility alterations within T cells, ultimately leading to T-cell exhaustion. KC, Kupffer cells; B, B cells; T, T cells; M, macrophages; TAM, tumor-associated macrophages; NK, natural killer; PD-L1, programmed death-ligand 1; CTLA-4, cytotoxic T-lymphocyte-associated protein 4; Treg, regulatory T; DC, dendritic cell; (Figure created with BioRender.com).

**Figure 2:**
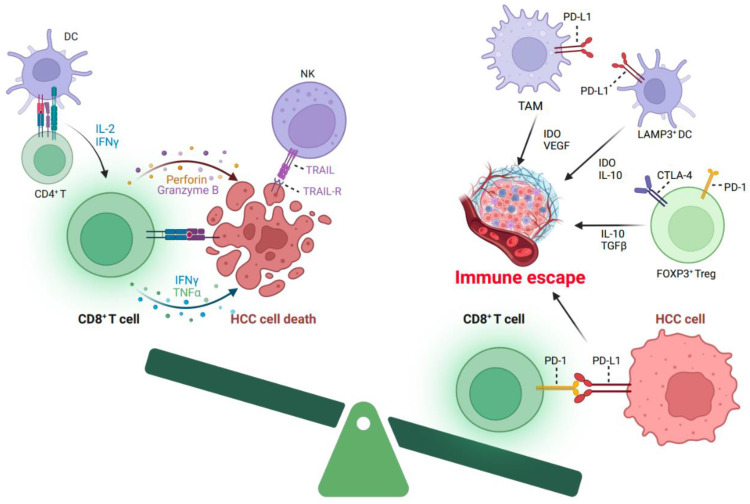
Key immune cell types and their roles in the HCC immune microenvironment. In the context of the HCC immune microenvironment, immune cells can be broadly categorized into two distinct types. The first group includes immune cells capable of recognizing tumor cells and initiating cytotoxic responses against malignant cells, while the second group comprises cells that exert immunosuppressive functions, ultimately leading to immune evasion. When the influence of the second group surpasses that of the first group, the immune microenvironment becomes conducive to tumorigenesis. This figure illustrates the pivotal cellular factors involved in these diverse activities. DC, dendritic cell; IDO, indoleamine 2,3-dioxygenase; NK, natural killer; TRAIL, tumor-necrosis-factor-related apoptosis-inducing ligand; TRAIL-R, tumor-necrosis-factor-related apoptosis-inducing ligand receptor; TAM, tumor-associated macrophage; PD-1, programmed cell death protein 1; PD-L1, programmed death-ligand 1; CTLA-4, cytotoxic T-lymphocyte-associated protein 4; Treg, regulatory T; VEFG, vascular endothelial growth factor. (Figure created with BioRender.com).

In the context of HCC, the microenvironment encompasses a dynamic interplay between immune cells, endothelial cells, fibroblasts, lymph vessels, and the ECM. Recent findings [14] indicate that the non-immune constituents of the tumor microenvironment, namely, cancer-associated fibroblasts (CAFs) and liver sinusoidal endothelial cells (LSECs), have been implicated in tumor immune evasion and the promotion of tumorigenesis. Originating from HSCs, CAFs play a significant role in fostering tumor-promoting inflammation by secreting elevated levels of IL-6, hepatocyte growth factor (HGF), vascular endothelial growth factor (VEGF), and angiopoietin-1 (Figure 1). This secretion, in turn, enhances the stem-cell-like characteristics of HCC cells and facilitates angiogenesis. Furthermore, LSECs, functioning as non-myeloid antigen-presenting cells (APCs), are capable of cross-presenting soluble exogenous antigens to CD8+ T cells, leading to the establishment of CD8+ T-cell tolerance [15]. Previous studies have demonstrated the infiltration of cytotoxic T lymphocytes (CTLs) and T regulatory lymphocytes (Tregs) within HCC tumors [16,17]. The functional roles of these distinct T lymphocyte populations are closely associated with their effects on anti-tumorigenesis. CTLs exhibit the ability to recognize tumor cells and subsequently engage in the release of cytotoxic enzymes and cytokines, leading to the elimination of malignant cells (Figure 2). Conversely, Tregs play a crucial role in promoting immunosuppression (Figure 2), which can potentially impede antitumor immune responses [18]. Previous research has confirmed the significance of a distinct prognostic factor for overall survival (OS) and disease-free survival (DFS) in HCC. This factor is characterized by a decreased presence of Tregs along with an increased presence of CTLs [19]. Additionally, it has been observed that the density of Tregs exhibits a correlation with HCC invasiveness, including the absence of tumor encapsulation and the presence of vascular invasion. NK cells are integral to the immune surveillance against tumors, playing a pivotal role. However, in HCC, the diminished frequency and compromised functionality of NK cells become contributing factors to cancer evasion. Within the TME, diverse cellular components, including CAFs, myeloid-derived suppressor cells (MDSCs), and tumor-associated macrophages (TAMs), produce immunosuppressive factors such as PGE2, IDO enzymes, and IFN-γ, which actively impede NK cell activation and cytotoxic activity [20]. Macrophages and neutrophils exert notable influence within the TME of HCC [21,22]. These distinct cell types can be categorized into two contrasting functional subsets, respectively. M1 macrophages and N1 neutrophils exhibit an anti-tumorigenic effect by producing antitumor factors and strengthening the antitumor immune response. In stark contrast, M2 macrophages and N2 neutrophils elicit entirely opposing effects. Metabolic byproducts originating from HCC tumors have the potential to exert adverse effects on T cells. A preceding investigation substantiated the role of HCC-released metabolites, including S-adenosyl-L-methionine (SAM) and methylthioadenosine (MTA), in inducing chromatin accessibility alterations within T cells (Figure 1), consequently culminating in T-cell exhaustion [23]. According to recent studies [24,25], the immune landscape of HCC has been categorized into three distinct levels. Notably, the “immune strong” subtype, characterized by a pronounced presence of γδ T cells and B-/plasma cells, has shown a significant association with improved prognosis. Conversely, the “immune low” subtype exhibits heightened infiltration of immunosuppressive cells and molecules, such as PD-1, PD-L1, and CTLA-4. The substantial heterogeneity observed in the immune microenvironment of HCC, both intra- and inter-tumor, emphasizes the significance of precise immune classification. This classification holds potential to greatly aid clinicians in their therapeutic decision-making processes, specifically in selecting the most suitable immunotherapy regimen for individual patients. Additionally, Ma et al. [26] investigated the relationship between intra-tumoral heterogeneity (ITH) and survival outcomes. Through the classification of tumors into high- or low-transcriptomic diversity score groups based on the average expression of 10 cancer stemness genes, they discovered that tumors with higher ITH scores exhibited poorer mOS and demonstrated a more diverse TME.

## 3. The Characteristics of T Cells in HCC

Owing to the immunosuppressive mechanisms operating within the TME, tumor-infiltrating lymphocytes (TILs), particularly CD8+ T cells, encounter challenges in their ability to effectively combat cancer cells (Figure 2). This includes the upregulation of PD-1, a known indicator of T-cell exhaustion, which contributes significantly to T-cell dysfunction. Notably, recent advancements in ICB therapies have presented promising prospects within the realm of antitumor interventions. Being consistent with this, anti-PD-1/PD-L1/CTLA-4 treatments have demonstrated remarkable efficacy across various cancer types, encompassing non-small-cell lung cancer (NSCLC) [27,28,29], melanoma [30,31], renal cell carcinoma [32,33], urologic cancer [34], and HCC [35,36]. However, the objective response rates observed in these clinical trials range from 20% to 58%. Despite advancements in immune therapy, a significant proportion of patients fail to achieve durable responses, necessitating an exploration of underlying mechanisms. Notably, emerging evidence suggests that additional inhibitory pathways within T cells may contribute to this resistance. Building upon this, Wherry et al. [37] conducted a study investigating T-cell exhaustion during chronic lymphocytic choriomeningitis virus (LCMV) infection, identifying three key characteristics: (1) upregulation of immune checkpoints, including PD-1, CTLA-4, and Lag-3; (2) impaired effector functions, characterized by deficient IFN-γ and TNF-α production; and (3) dysregulated expression of genes involved in cellular movement, encompassing chemotaxis, adhesion, migration, and exhibiting distinct transcription factor profiles. Importantly, these alterations have been corroborated in studies focusing on T cells in cancer contexts [38,39,40]. As the majority of current immunotherapies center on the targeting of immune checkpoints or the genetic modification of a patient’s T- cells to carry chimeric antigen receptors, facilitating these T cells in more effectively recognizing and combatting specific cancer cell surface antigens, it becomes evident that the proper functioning of a patient’s T cells is paramount for the success of immunotherapy. In the absence of proper T-cell functionality during immunotherapy, the treatment will likely prove ineffective, even when employing immune checkpoint inhibitors or genetic modifications of the patient’s immune cells. Therefore, it becomes apparent that comprehending T-cell exhaustion plays a pivotal role in gaining insights into the underlying mechanisms that result in the ineffectiveness of immunotherapy. This understanding forms a robust foundation for further exploration and the development of therapeutic strategies in this domain.

While previous studies have identified some shared characteristics between T cells in chronic virus infection and tumor contexts, recent investigations have revealed distinct differences, including alterations in the expression patterns of transcription factors [41,42]. Notably, the transcriptional profiles of T cells in cancer exhibit significant changes; however, uncovering the primary regulator responsible for driving the dysfunction process presents a complex challenge. This complexity arises due to the involvement of numerous transcription factors and gene signatures that overlap with those observed in normal activated T cells [43]. Moreover, there is concurrent expression of immune checkpoints, such as Lag-3, Tim-3, and TIGIT, on dysfunctional T cells as well as on recently activated T cells [44]. These findings emphasize the need for further research to elucidate the precise mechanisms underlying T-cell dysfunction in both chronic virus infection and cancer, considering the intricate interplay of transcription factors and the shared expression patterns of immune checkpoints.

In addition to genetic factors, T-cell dysfunction in cancer is closely associated with substantial metabolic alternations. Under normal conditions, T cells predominantly depend on oxidative phosphorylation (OXPHOS) and mitochondrial fatty acid oxidation as their primary pathways to generate ATP. However, dysfunctional T cells exhibit a persistent loss of mitochondrial function and dysregulation of glucose metabolism pathways, including impaired glucose uptake and utilization [45]. The high energy consumption of a tumor leads to a sharp reduction in essential nutrients, which generates an unfavorable metabolic microenvironment characterized by low energy, low oxygen and high lactic acid. These metabolic alterations contribute to the compromised functionality of T cells in the TME.

Research aligned with these findings has demonstrated that interventions targeting the production of intermediates in the glucose metabolic pathway, along with increasing amino acids (such as glutamine, tryptophan, and Kynurenine) and neutralizing acidic components, exert significant influences on intra-tumoral T-cell functions [46,47,48]. Notably, Ho et al. reported that elevating the glycolytic metabolite phosphoenolpyruvate (PEP) to sustain Ca^2+^-NFAT signaling can effectively restore effector functions of T cells [48]. These insights highlight the potential of metabolic modulation as a promising avenue for therapeutic interventions to enhance T-cell responses within the TME, thus bolstering efforts to improve cancer immunotherapy efficacy.

## 4. Exploring T-Cell States in HCC through ScRNA-seq and Spatial Analysis

In light of the pronounced heterogeneity of the tumor immune microenvironment in HCC, it becomes imperative to leverage innovative technologies, including mass cytometry, scRNA-seq, and spatial analysis, for a thorough exploration of the diverse spectrum of T-cell states in HCC, since this heterogeneity encompasses variations in the type, abundance, and functional states of immune cells, as well as their interactions within the surrounding microenvironment [49,50]. To better comprehend the intricacies of tumor microstructures in HCC, recent studies have effectively employed these advanced techniques [51,52]. However, before delving into the details of these findings, it is crucial to introduce scRNA-seq technology and its capabilities. ScRNA-seq allows for the high-resolution examination of individual cells’ gene expression profiles, offering insights into the heterogeneity and functional states of T cells within the HCC immune microenvironment.

Single-cell sequencing holds immense promise in advancing tumor diagnosis, treatment, and prognosis prediction through comprehensive profiling of various omics, including genomics, transcriptomics, proteomics, epigenomics, and metabolomics sequencing [53,54]. Among these cutting-edge methodologies, scRNA-seq emerges as a pivotal technique, empowering researchers to delve into the complex cellular composition, molecular landscape, and dynamic interactions between tumor cells and immune cells [55,56]. By offering a high-resolution view of individual cells, scRNA-seq provides unprecedented insights into the heterogeneity and functional states of diverse cell populations within the tumor microenvironment [57,58]. Several investigations have been conducted to explore the heterogeneity of immune cells and tumor cells in primary HCC [59,60,61]. Ho DW et al. conducted a comprehensive study on a cohort of eight HCC cases, each comprising approximately 1000 cells on average, with hepatitis B viral infection as the underlying etiology [59]. Utilizing scRNA-seq analysis, the authors uncovered a substantial enrichment of TAMs, and this was inversely correlated with T-cell abundance. Particularly noteworthy, the study established a statistically significant correlation between the expression of CD163, a marker for M2 macrophages, and LAIR1 not only within this cohort but also across other datasets, including TCGA-LIHC RNA-seq. This finding suggests a potential immunosuppressive role of LAIR1 in M2 macrophages. Additionally, the researchers characterized a co-inhibitory signal, TIGIT–NECTIN2, between complementary T cells and APCs. Immunohistochemistry analyses further confirmed the overexpression of TIGIT–NECTIN in HCC, independent of non-HCC and cirrhotic livers resulting from HBV infection, underscoring the preferential utilization of the TIGIT–NECTIN2 axis as a tumor evasion strategy and its pivotal role in shaping the immunosuppressive tumor microenvironment in HCC.

To explore the single-cell landscape of the tumor immune microenvironment in early-relapse HCC, Sun et al. conducted a comprehensive analysis of single-cell transcriptomes obtained from 6 primary HCC patients and 12 early-relapse HCC patients [62]. The findings revealed distinct immunological landscapes in early-relapse HCC compared to primary HCC. Notably, the ecosystem of early-relapse HCC exhibited reduced levels of Tregs and increased fractions of CD8+ T cells. Interestingly, the heightened CD8+ T-cell population observed in early-relapse tumors exhibited an aberrant functional state. This state was marked by CD161 overexpression, low cytotoxicity, restricted clonal expansion, and innate-like characteristics, all of which were associated with unfavorable prognostic outcomes. Furthermore, these evasion mechanisms appeared to impede dendritic cells (DCs) from effectively activating CD8+ T cells. These findings shed light on the complexities of the tumor immune microenvironment in early-relapse HCC and have implications for understanding the underlying mechanisms governing immune escape and prognosis in this clinical context.

While scRNA-seq enables the identification of cell subpopulations within the tumor, it falls short in providing insights into the spatial structures and local interactions between tumor and immune cells in their native environment [63]. Given the spatial organization inherent to the liver, the correlation of RNA profiles with cell positioning in situ assumes significant relevance [64]. In response to this requirement, the incorporation of direct spatial transcriptomics techniques emerges as a promising avenue for advancing our understanding of the complex immune landscape within HCC. For instance, Liu et al. employed spatial transcriptomics and scRNA-seq to identify a unique spatial organization termed the “tumor immune barrier” (TIB) within the HCC TME, which has been associated with the efficacy of immune therapies [65]. The unique spatial organization in the tumor microenvironment involving SPP1+macrophages and cancer-associated fibroblasts near the tumor boundary significantly impacts the effectiveness of immunotherapy in HCC patients receiving anti-PD-1 treatment. This TIB structure restricts immune cell infiltration into the tumor core, reducing immunotherapy efficacy.

To meet the demand for novel therapeutic interventions in HCC, scRNA-seq plays a pivotal role not only in identifying potential target molecules but also in advancing tumor classification, thereby enabling the development of more personalized and precise therapeutic strategies for diverse patient cohorts. Already, scRNA-seq has contributed to the establishment of HCC classifications, facilitating prognostic prediction and aiding treatment decision-making [25]. However, further prospective validation of these classifications is warranted.

## 5. Immune Treatment Options Targeting Reactivation of Disfunction T Cells in HCC

**Figure 3:**
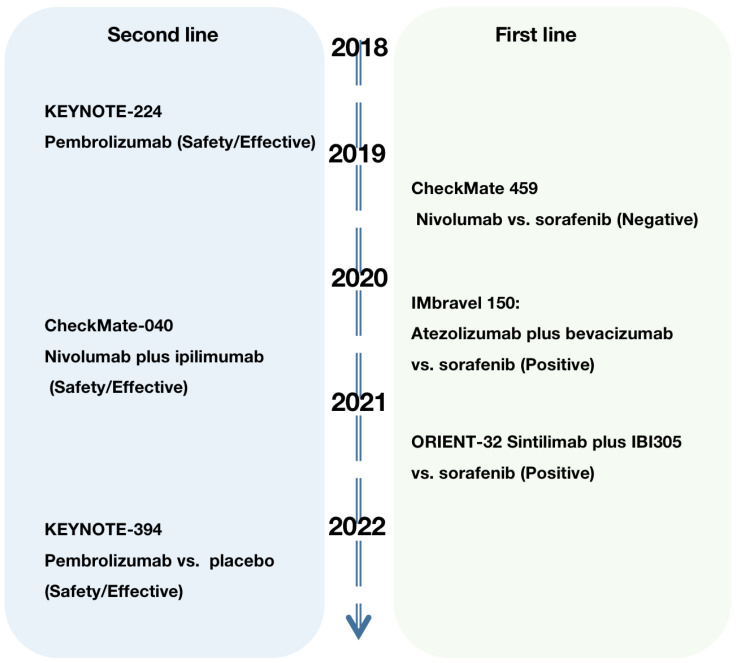
Several important trials in the development of immune therapy for HCC.

The association of HCC development with immune tolerance and suppression has prompted the exploration of therapies targeting dysfunctional T-cell reactivation, showing great promise. As previously discussed, besides PD-1, dysfunctional T cells also display upregulation of other immune inhibitory checkpoints, including CTLA-4, Lag-3, Tim-3, and TIGIT. Consequently, an increasing number of studies have focused on investigating the potential roles of immune checkpoint inhibitors (ICIs) in HCC. Over the last five years, the introduction of ICIs, including pembrolizumab and nivolumab targeting PD-1, durvalumab and atezolizumab targeting PD-L1, and tremelimumab and ipilimumab targeting CTLA-4, has marked the beginning of a transformative era in HCC management. PD-1 and its ligand PD-L1 play critical roles as negative regulators of the immune system. These immune checkpoint molecules can be expressed not only in T cells but also APCs (such as DCs and TAMs) and tumor cells. PD-1 was originally identified and cloned by Honjo T et al. in 1992 [66], whereas PD-L1 (also known as B7-H1) was subsequently recognized as a potential mechanism of immune evasion by Lieping Chen et al. in 2002 [67]. Later, Topalian SL et al. substantiated the safety and efficacy of anti-PD-1 antibody in cancer treatment in 2012 [68]. Mechanistically, the binding of PD-1 and PD-L1 leads to dephosphorylation of proximal T-cell receptor (TCR) signaling molecules [69], ultimately resulting in T-cell inactivation. In a related context, it is worth noting that another critical immune checkpoint, CTLA-4, expressed on activated T cells, also plays a pivotal role in regulating immune responses. CTLA-4 interacts with the coinhibitory ligands B7 (CD80 and CD86) present on antigen-presenting cells (APCs) [70]. This interaction inhibits T-cell activation and proliferation, preventing excessive immune responses and autoimmune reactions by suppressing the PI3K-Akt pathway (which is a vital signaling pathway for T-cell activation) [71]. Moreover, it is noteworthy that the binding affinity of coinhibitory molecules, such as CTLA-4 on T cells with CD80 and CD86 on APCs, surpasses that of costimulatory molecules, such as CD28 on T cells with CD80 and CD86 on APCs [72]. This disparity in affinity has significant implications for the regulation of T-cell activation and immune responses. As a consequence, anti-CTLA-4 therapies boost the abundance and activation of CD4+ and CD8+ T cells [73], enhancing their antitumor responses. In this context, we provide a comprehensive overview of ICI-based trials, combinations of ICI-based therapies (as indicated in Table 1), and the key trials that are instrumental in advancing immunotherapy for HCC, as illustrated in Figure 3.

## 6. Monotherapies of ICIs

Several clinical trials have investigated the use of ICIs as monotherapy in HCC (Table 1). To elucidate the role of nivolumab in advanced HCC, two related clinical trials were conducted sequentially: the non-comparative, open-label phase I/II CheckMate 040 trial [35] and the phase III randomized controlled trial CheckMate 459 (nivolumab vs. sorafenib) [74]. In CheckMate 040, nivolumab demonstrated an ORR of 15% (95% CI, 6–28) in the dose-escalation phase and exhibited a manageable safety profile. Consequently, the FDA approved nivolumab as a second-line treatment for advanced HCC patients who had received prior treatment with sorafenib. However, the subsequent CheckMate 459 trial did not meet the primary overall survival (OS) endpoints in both first- and second-line settings, leading to the withdrawal of nivolumab monotherapy’s indication from the US market. In the phase II trial KEYNOTE-224 [75], pembrolizumab, another anti-PD-1 antibody, demonstrated favorable efficacy and safety profiles in advanced HCC patients, with an observed ORR of 17% (95% CI, 11–26). Subsequently, in the randomized, double-blind, and phase III trial KEYNOTE-240 [76], pembrolizumab exhibited promising results with a median OS of 13.9 months compared to 10.6 months for placebo (HR 0.781, 95% CI 0.611 to 0.998; *p* = 0.0238). However, it is worth noting that the improvements in OS and PFS did not meet the predefined statistical significance criteria (the *p*-value did not exceed 0.0174). It is noteworthy that a similar trial, KEYNOTE-394 [77], yielded a positive outcome, showing a superior median OS with pembrolizumab versus placebo (14.6 months versus 13.0 months; HR 0.79, 95% CI 0.63–0.99; *p* = 0.0180). Despite ongoing debates surrounding these two clinical trials [83], the data collectively demonstrate the activity of pembrolizumab in the treatment of HCC.

## 7. Combination Therapies

**Figure 4:**
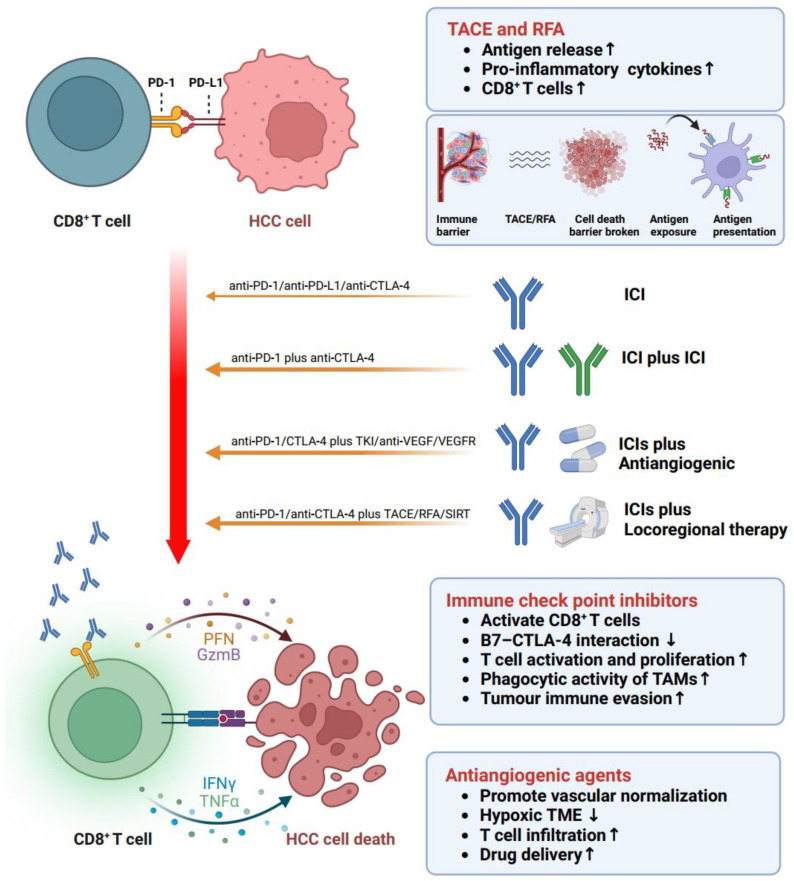
Synergistic effects of combined therapies. The concurrent use of ICIs alongside antiangiogenic agents or locoregional therapy has been shown to transition the immunosuppressive TME into an immune-active state. This phenomenon is mediated through several interconnected mechanisms, which include enhanced antigen exposure, leading to an improved antigen presentation process. Additionally, the combination therapy facilitates vascular normalization, thereby enhancing drug delivery and promoting the infiltration of T cells into the tumor site. Moreover, the activation of CD8+ T cells and other crucial antitumor immune cells further contributes to the observed synergistic effects, culminating in a robust and coordinated anticancer immune response. (Figure created with BioRender.com).

It is noteworthy that monotherapy with ICIs in HCC demonstrates a relatively lower response rate (approximately 20%) compared to melanoma and NSCLC (approximately 50%). As a result, combination therapies, such as ICIs in conjunction with antiangiogenic agents or locoregional therapy, have emerged as a promising strategy (Figure 4). These approaches hold the potential to enhance treatment efficacy and overcome the challenges posed by immune escape and resistance mechanisms in HCC, thereby offering new avenues for improving patient outcomes.

### 7.1. ICIs + ICIs

Certainly, in light of the distinct functional roles exhibited by PD-1/PD-L1 and CTLA-4 across various stages of the immune response, the concurrent co-administration of PD-1/PD-L1 inhibitors and CTLA-4 inhibitors holds promise in the context of potentially yielding complementary and synergistic effects [84]. To explore this therapeutic approach, a randomized phase I/II study (NCT02519348) [78] was conducted, investigating the safety and efficacy of tremelimumab (a CTLA-4 inhibitor) in combination with durvalumab (a PD-L1 inhibitor) in unresectable HCC patients. The study revealed a confirmed ORR of 24% (95% CI, 14.9 to 35.3) for the combination therapy. Furthermore, the median OS reached 18.7 months (95% CI, 0.8 to 27.3), accompanied by an observed manageable safety profile. Following these promising results, an open-label, controlled, phase III trial (NCT03298451) [80] was initiated to compare the safety and effectiveness of the combined therapy (tremelimumab plus durvalumab) with the first-line treatment sorafenib in the treatment of unresectable HCC patients. The combination therapy demonstrated encouraging outcomes, as the OS at 36 months was 30.7%, in contrast to 20.2% for sorafenib (HR 0.78, 96.02% CI 0.65 to 0.93; *p* = 0.0035).

The OS rate for the durvalumab monotherapy group was 24.7%, indicating non-inferiority in comparison to the sorafenib-treated group, which exhibited an OS rate of 20.2% (HR 0.86; 95.67% CI, 0.73 to 1.03; non-inferiority margin, 1.08). Furthermore, the exploration of alternative combination therapies has yielded encouraging results. Notably, the combination of ipilimumab (a CTLA-4 inhibitor) with nivolumab (a PD-L1 inhibitor) in the CheckMate 040 trial has demonstrated significant promise. In arm A of the trial, which involved administering 4 doses of ipilimumab at 3 mg/kg alongside nivolumab at 1 mg/kg every 3 weeks, followed by subsequent nivolumab dosing at 240 mg every 2 weeks, an impressive ORR of 32% (95% CI, 20–47) was observed [79]. These compelling findings led to the accelerated approval of this combination therapy in the US.

### 7.2. ICIs + Antiangiogenic Agents

In addition to exploring the synergy between ICIs, recent scholarly investigations have shifted their attention toward the prospects offered by combining ICIs with antiangiogenic agents as a therapeutic approach for HCC. Antiangiogenic drugs primarily target vascular endothelial growth factor receptor (VEGFR) or VEGF, encompassing tyrosine kinase inhibitors (TKIs) such as sorafenib, lenvatinib, and regorafenib, as well as anti-VEGF monoclonal antibodies like bevacizumab. These agents have been shown to promote vascular normalization and enhance immune responses in HCC [85,86]. By regulating tumor vessels, they alleviate the hypoxic tumor microenvironment and facilitate T-cell infiltration. The promising results emerged from an open-label, phase 1b trial (GO30140) [81], where the combination of atezolizumab and bevacizumab demonstrated a longer median PFS compared to atezolizumab alone (5.6 months vs. 3.4 months, HR 0.55, 80% CI 0.40–0.74; *p* = 0.011) in patients with unresectable HCC. Building on this encouraging anti-HCC activity, the well-known, global, open-label, phase III trial IMbravel150 further compared the combination of atezolizumab and bevacizumab with the first-line treatment of sorafenib in patients with unresectable HCC [82]. The final analysis yielded significant outcomes, showing a hazard ratio (HR) for death with the combination versus sorafenib (HR 0.58, 95% CI 0.42–0.79; *p* < 0.001). Moreover, the median PFS of atezolizumab plus bevacizumab (6.8 months) was superior to that of sorafenib (4.3 months) (HR 0.59; 95% CI, 0.47–0.76; *p* < 0.001), and a better OS (OS at 12 months: 67.2% vs. 54.6%) was achieved. Based on the success of IMbravel150, the FDA approved the combination of atezolizumab plus bevacizumab as a first-line treatment for advanced HCC patients. In addition to the successful IMbravel150 trial, another study with a similar design, ORIENT-32 [36], was conducted as a randomized, open-label investigation. This study aimed to compare the combination of bevacizumab biosimilar IBI305 (a monoclonal antibody targeting VEGF) with sintilimab (a PD-1 inhibitor) against sorafenib in patients with unresectable HBV-associated HCC. The ORIENT-32 trial also yielded significant results, demonstrating improved OS (HR 0.57, 95% CI 0.43–0.75; *p* < 0.0001) and PFS (4.6 months vs. 2.8 months; HR 0.56, 95% CI 0.46–0.70; *p* < 0.0001).

### 7.3. ICIs + Locoregional Therapy

Research has elucidated the existence of a tumor-associated immune barrier encompassing the neoplastic tissue, presenting a substantial impediment to the effectiveness of immunotherapeutic interventions [87]. This immune barrier functions by impeding the antigen presentation process, thereby restricting the activation of antitumor immune responses [65]. Indeed, it is widely recognized that locoregional therapies, such as tumor ablation, TACE, or radiotherapy, can enhance the antigen presentation process by causing tumor cell or structure destruction. Hence, the amalgamation of ICIs with locoregional therapeutic modalities exhibits potential as a viable approach for managing HCC at intermediate or advanced stages. Several clinical trials have demonstrated the safety and efficacy of ICIs combined with radiofrequency ablation (RFA) or TACE. In the NCT01853618 trial [88], patients who received combined therapy with tremelimumab and ablation exhibited a significant increase in CD8+ T cells in the six-week tumor biopsies. Similarly, in a phase 1b study (NCT03397654) [89], patients with intermediate-stage HCC who underwent two rounds of conventional TACE followed by pembrolizumab showed no synergistic toxicity from the combination, and no dose-limiting toxicities were reported.

Numerous ongoing investigations are currently underway within this domain, encompassing research into the synergistic potential of ICIs, such as durvalumab plus tremelimumab, in conjunction with TACE or RFA for HCC or biliary tract cancer (NCT02821754). Furthermore, studies are being conducted on nivolumab in combination with TACE for advanced HCC (NCT03143270), as well as on the utilization of selective internal radiation therapy (SIRT) in tandem with pembrolizumab (NCT03099564) or nivolumab (NCT02837029) [90]. However, it should be noted that locoregional therapy may induce hypoxia, enhance vascular permeability, and increase levels of vascular endothelial growth factors (VEGFs) and transforming growth factor-beta (TGFβ), ultimately leading to immune suppression. Therefore, the addition of antiangiogenic drugs may further enhance the combined effect of ICIs and locoregional therapy [91]. A randomized, double-blind, multicenter, phase III study (NCT03778957) is currently underway to investigate the efficacy and safety of TACE in combination with durvalumab plus bevacizumab, compared to TACE plus durvalumab, in patients with locoregional HCC.

## 8. CAR-T and TCR-T

In addition to ICIs, several innovative immunotherapies have emerged, including adoptive cell therapy (ACT), chimeric antigen receptor (CAR)-T cell, and T-cell receptor (TCR)-engineered T-cell therapy. These therapies aim to target tumor-associated antigens (TAA) to combat tumor cells. The expression of these TAA is associated with patients’ prognosis [92], and some, such as alpha-fetoprotein (AFP) and PRL3, are exclusively expressed in tumors rather than in healthy adult tissues. Targeting TAAs in therapies may enhance the immune response. Regarding HCC [13], key TAAs include P53, hTERT, glypican-3 (GPC-3), AFP, and others.

Preclinical studies have demonstrated the efficacy of CAR-T cells in mouse models. Makkouk A et al. [93] developed GPC-3.CAR/sIL-15 Vδ1 T cells targeting GPC-3 and capable of expressing and secreting IL-15. The results showed that these CAR-T cells proliferated within the tumor and controlled tumor growth in a HepG2 mouse model. Emerging clinical trials evaluating the safety and efficacy of CAR-T cells in HCC patients have been conducted in recent years. Currently, there are at least 15 phase I/II clinical trials recruiting HCC patients to test CAR-T cells (ClinicalTrials.gov, October 2023). Five trials targeting GPC-3 have been completed, including NCT03884751, NCT03146234, NCT03980288, NCT02905188, and NCT02395250; however, as of October 2023, no study results have been posted on ClinicalTrials.gov. Another CAR-T-cell therapy tested in three solid tumors, including advanced HCC, is the IL-7- and CCL19-secreting CAR-T cell, as registered in NCT03198546 [94]. This phase I clinical trial evaluated IL-7- and CCL19-secreting CAR-T cells in patients with GPC3 or mesothelin (MSLN) expression. In this trial, a patient with HCC who received anti-GPC3-7 × 19 CAR-T treatment achieved complete tumor regression. However, to date, there are no approved CAR-T-cell therapies for solid tumors, primarily due to the non-MHC-restricted manner, heterogeneity, immunosuppressive TME, and CAR-T-cell trafficking or infiltration challenges [95]. Future research directions may involve using CAR-T cells as part of combination therapies for HCC or discovering specific TAAs for HCC. Compared to CAR-T-cell therapy, TCR-T-cell therapy can exhibit superior anti-solid tumor capabilities. However, the application of TCR-T-cell therapy is limited due to its dependence on the MHC-dependent antigen presentation system. Currently, at least seven phase I/II or observational clinical trials are recruiting HCC patients for TCR-T-cell therapy (ClinicalTrials.gov, October 2023). In this context, a phase I/II multicenter trial (NCT05417932) is evaluating SCG101 in subjects with HBV-related HCC. SCG101 is also being tested in combination with PD-1/PD-L1 ICIs in HBV-related HCC in a phase I trial (NCT05339321).

## 9. Conclusions

Known for its ability to counteract exaggerated immune responses triggered by a wide range of environmental factors, the liver stands out as an organ capable of immune tolerance. However, disruptions to this delicate immune equilibrium, whether caused by viral infections or other forms of injury, initiate a series of events characterized by chronic inflammation and the accumulation of immune cells within the liver. Critical roles in driving the progression of this immunosuppressive microenvironment are played by both immunosuppressive cells and non-immune components. Within this TME, the prevalence of immunosuppressive mechanisms poses a significant challenge for TILs, with T cells in particular encountering obstacles in their ability to effectively engage with cancer cells. The upregulation of immune checkpoints, most notably PD-1, CTLA-4, and Lag-3, combined with the absence of antitumor cytokines, distinct profiles of transcription factors, and marked metabolic shifts, results in an environment characterized by reduced energy, insufficient oxygen levels, and elevated concentrations of lactic acid. These factors collectively lead to the eventual exhaustion of T cells. Collectively, these findings emphasize the potential transformative influence of immune therapies designed to revive malfunctioning T cells, holding the promise of fundamentally altering the landscape of HCC management. Investigations have substantiated the significance of ratios of immune checkpoint expressions in shaping the outcomes of ICIs interventions. The urgency to develop techniques capable of accurately identifying patients poised to benefit from immune therapies remains a critical priority. The integration of scRNA-seq and spatial analysis holds the promise of providing comprehensive insights into tumor heterogeneity and identifying viable targets for therapeutic intervention.

While immune therapies have demonstrated their effectiveness against various solid tumors, the sole utilization of ICIs in HCC still faces a relatively moderate response rate. This reality highlights the importance of integrated approaches involving ICIs, antiangiogenic agents, and locoregional therapies as a leading avenue of development. The encompassing barrier surrounding HCC lesions significantly hampers the infiltration of immune cells and diminishes the efficacy of ICIs. However, locoregional therapies hold the potential to dismantle this obstacle, enabling the ICIs and antiangiogenic agents to reignite the tumor-responsive abilities of T cells. This convergence ultimately leads to the elimination of cancerous cells and a notable improvement in patient prognoses. Innovative immunotherapies, such as CAR-T-cell and TCR-T-cell therapy, target TAAs linked to patient prognosis, and despite facing challenges, they continue to show promise in both preclinical and clinical studies. Future research endeavors could focus on delving more deeply into the mechanisms governing the formation of tumor barriers and the intricate microstructural characteristics involved. This could unveil an expanded array of therapeutic targets amenable to sequential interventions. Without a doubt, the convergence of diverse treatment modalities, combined with the ongoing advancement of scientific exploration, holds the promise of ushering in a progressively refined paradigm for the care of HCC patients.

## Figures and Tables

**Table 1:** Outcomes from pivotal clinical trials evaluating immunotherapeutic approaches for advanced-stage HCC.

Trials/Phase	Study Period	Treatments	N	ORR	MOS (mo) (HR, 95% CI)	MPFS (mo) (HR, 95% CI)	Treatment-Related Adverse Events		Result
							Grades 3–5	Leading to Death	
Monotherapies of ICIs									
CheckMate040/I II /NCT01658878 [35]	2012-11-26 to 2016-08-08	Nivolumab/0.3 mg/kg (dose expansion)	262	15%	Not available	Not available	25%	0	Safety/Effective
CheckMate459/III/ NCT02576509 [74]	2016-01-11 to 2017-05-24	Nivolumab (240 mg every 2 weeks) vs. sorafenib (400 mg twice daily)	743	15% vs. 7%; *p* = NA	16.4 vs. 14.7 (HR 0.85, 0.72–1.02; *p* = 0.075)	3.7 vs. 3.8 (HR 0.93, 0.79–1.10, NS)	22% vs. 49%	1% vs. 0.3%	Negative
KEYNOTE-224/II/NCT02702414 [75]	2016-06-07 to 2017-02-09	Pembrolizumab (200 mg every 3 weeks)	169	17%	12.9 (9.7–15.5)	4.9 (3.4–7.2)	25%	1%	Safety/Effective
KEYNOTE-240/III/NCT02702401 [76]	2016-05-31 to 2017-11-23	Pembrolizumab (200 mg every 3 weeks) vs. placebo	413	18.3% vs. 14.4% (*p* = 0.00007)	13.8 vs. 10.6 (0.78, 0.61–1.00; *p* = 0.024)	3.0 vs. 2.8 (HR 0.72, 0.57–0.90; *p* = 0.002)	52.7% vs. 46.3%	2.5% vs. 3.0%	Negative
KEYNOTE-394/III/NCT03062358 [77]	2017-05-31 to 2019-12-11	Pembrolizumab (200 mg every 3 weeks) vs. placebo	453	12.7% vs. 1.3% (*p* < 0.0001)	14.6 vs. 13.0 (0.79, 0.63–0.99; *p* = 0.0180)	2.6 vs. 2.3 (HR 0.74, 0.60–0.92; *p* = 0.0032)	14.3% vs. 5.9%	0 vs. 0	Positive
Combination therapies of ICIs									
NCT02519348/I II [78]	2015-08-10 to 2020-02-28	Tremelimumab + durvalumab/T300 + D	74	24.0%	18.7 (10.8 to 27.3)	2.17 (1.91 to 5.42)	37.8%	1.4%	Safety/Effective
		Durvalumab (1500 mg every 4 weeks)	104	10.6%	13.6 (8.7 to 17.6)	2.07 (1.84 to 2.83)	20.8%	2.9%	
		Tremelimumab (750 mg every 4 weeks)	69	7.2%	15.1 (11.3 to 20.5)	2.69 (1.87 to 5.29)	43.5%	0	
		Tremelimumab + durvalumab/T75 + D	84	9.5%	11.3 (8.4 to 15.0)	1.87 (1.77 to 2.53)	24.4%	1.2%	
CheckMate 040/I II/ NCT01658878 [79]	2016-01 to 2019-01	Arm A nivolumab 1 mg/kg + ipilimumab 3 mg/kg every 3 weeks, followed by nivolumab 240 mg every 2 weeks	148	32%	Not available	Not available	53.0%	2.0%	Safety/Effective
NCT03298451/III [80]	2017-11 to 2019-06	Tremelimumab (300 mg one dose) + durvalumab (1500 mg every 4 weeks)	393	20.1%	OS (vs sorafenib) HR 0.78 (96.02% CI, 0.65 to 0.93; *p* = 0.035)	3.78 (3.68–5.32) *p* = NS	50.5%	2.3%	Positive
		Durvalumab (1500 mg every 4 weeks)	389	17.0%	OS (vs. sorafenib) HR 0.86 (95.67% CI, 0.73 to 1.03; non-inferiority margin, 1.08)	3.65 (3.19–3.75) *p* = NS	37.1%	0	
		Sorafenib (400 mg twice daily)	389	5.1%	MOS13.77 (12.25 to 16.13)	4.07 (3.75–5.49) *p* = NS	52.4%	0.8%	
Combination therapies of ICIs with antiangiogenic agents									
GO30140/Ib/ NCT02715531 [81]	2016-07-20 to 2018-07-31	Group A: Atezolizumab (1200 mg) + bevacizumab (15 mg/kg) every 3 weeks	104	36%	13.8–not estimable	7·3 (5·4–9·9)	53%	3%	Positive
		Group F: Atezolizumab (1200 mg) + bevacizumab (15 mg/kg) every 3 weeks	60	20%	8.3–not estimable	5.6 vs. 3.4 (HR 0.55, 80% CI 0.57–0.90; *p* = 0.002)	20%	0	
		Group F: Atezolizumab	59	17%	8.2–not estimable		5%	0	
IMbravel150/III NCT03434379 [82]	2018-03-15 to 2019-01-30	Atezolizumab (1200 mg) + bevacizumab (15 mg/kg) every 3 weeks	336	30% vs. 11% (*p* < 0.001)	19.2 vs. 13.4 (HR 0.66, 0.52–0.85; *p* = 0.0009)	6.8 vs. 4.3 (HR 0.65, 0.53–0.81; *p* = 0.0001)	36%	2%	Positive
		Sorafenib (400 mg twice daily)	165				46%	1%	
ORIENT-32/III NCT03794440 [36]	2019-02-11 to 2020-01-15	Sintilimab (200 mg) + IBI305 (15 mg/kg) every 3 weeks	380	20.5% vs. 4.1%, (*p* < 0.0001)	not estimable vs. 10.4 (HR 0.57,0.43–0.75; *p* < 0.0001)	4.6 vs. 2.8 (HR 0.56, 0.46–0.70; *p* < 0.0001)	33.7%	2%	Positive
		Sorafenib (400 mg twice daily)	191				35.7%	1%	

## Data Availability

The data can be shared up on request.

## Abbreviations

Hepatocellular carcinoma (HCC); overall response rate (ORR); progression-free survival (PFS); overall survival (OS); immune checkpoint inhibitor (ICI); Hepatitis B viruses (HBV); Hepatitis C viruses (HCV); alcoholic steatohepatitis (ASH); non-alcoholic steatohepatitis (NASH); non-alcoholic fatty liver disease (NAFLD); Barcelona Clinic Liver Cancer (BCLC); transarterial chemoembolization (TACE); immune checkpoint blockade (ICB); tumor microenvironment (TME); hepatic stellate cells (HSCs); extracellular matrix (ECM); single-cell RNA sequencing (scRNA-seq); cancer-associated fibroblasts (CAFs); liver sinusoidal endothelial cells (LSECs); hepatocyte growth factor (HGF); vascular endothelial growth factor (VEGF); antigen-presenting cells (APCs); cytotoxic T lymphocytes (CTLs); T regulatory lymphocytes (Tregs); disease-free survival (DFS); myeloid-derived suppressor cells (MDSCs); tumor-associated macrophages (TAMs); S-adenosyl-L-methionine (SAM); methylthioadenosine (MTA); intra-tumoral heterogeneity (ITH); tumor-infiltrating lymphocytes (TILs); non-small-cell lung cancer (NSCLC); dendritic cells (DCs); T-cell receptor (TCR); hazard ratio (HR); radiofrequency ablation (RFA); adoptive cell therapy (ACT); chimeric antigen receptor (CAR); tumor-associated antigens (TAA); alpha-fetoprotein (AFP); glypican-3 (GPC-3).
